# UPO Biobank: The Challenge of Integrating Biobanking into the Academic Environment to Support Translational Research

**DOI:** 10.3390/jpm13060911

**Published:** 2023-05-29

**Authors:** Valentina Bettio, Eleonora Mazzucco, Chiara Aleni, Silvia Cracas, Carmela Rinaldi, Annamaria Antona, Marco Varalda, Jacopo Venetucci, Daniela Ferrante, Antonio Rimedio, Daniela Capello

**Affiliations:** 1 UPO Biobank, University of Piemonte Orientale, 28100 Novara, Italy; 2 Department of Translational Medicine, Center of Excellence in Aging Sciences, University of Piemonte Orientale, 28100 Novara, Italy; 3 Department of Sustainable Development and Ecological Transition, University of Piemonte Orientale, 13100 Vercelli, Italy; 4 Learning and Research Area, A.O.U. Maggiore della Carità, 28100 Novara, Italy; 5 Ethics Committee of the University “Hospital Major of Charity” in Novara, Local Health Authorities Biella, 28100 Novara, Italy

**Keywords:** biobank, disease biobank, population biobank, precision medicine, translational research, sample storage, data storage, sample management

## Abstract

Biobanks are driving motors of precision and personalized medicine by providing high-quality biological material/data through the standardization and harmonization of their collection, preservation, and distribution. UPO Biobank was established in 2020 as an institutional, disease, and population biobank within the University of Piemonte Orientale (UPO) for the promotion and support of high-quality, multidisciplinary studies. UPO Biobank collaborates with UPO researchers, sustaining academic translational research, and supports the Novara Cohort Study, a longitudinal cohort study involving the population in the Novara area that will collect data and biological specimens that will be available for epidemiological, public health, and biological studies on aging. UPO Biobank has been developed by implementing the quality standards for the field and the ethical and legal issues and normative about privacy protection, data collection, and sharing. As a member of the “Biobanking and Biomolecular Resources Research Infrastructure” (BBMRI) network, UPO Biobank aims to expand its activity worldwide and launch cooperation with new national and international partners and researchers. The objective of this manuscript is to report an institutional and operational experience through the description of the technical and procedural solutions and ethical and scientific implications associated with the establishment of this university research biobank.

## 1. Introduction

Medicine is increasingly refining its ability to provide “custom-made” responses to patients and society. This personalized approach, based on the concept of the uniqueness of the person, enables the development of precision medicine, which helps to optimize prevention strategies and the most suitable therapies for each individual [1,2,3,4]. This paradigmatic shift in medicine is supported by the improvements in molecular investigations and bioinformatics, which allow the integrated analysis of a multiplicity of biological data with personal information (health, lifestyle, habits, socioeconomic, demographic, etc.), but imposes, at the same time, the need for a critical mass of biological samples and information collected according to the scientific criteria and high-quality standards [5,6,7]. Research biobanks have been created to meet these needs and, from the first appearance of the term biobank in a scientific report in 1996 [8], their diffusion and development have soared all over the world [9,10,11,12,13,14,15,16]. Biobanks can be properly defined as legal entities or part of a legal entity that performs, in a standardized way, the acquisition, storage, and distribution of high-quality biological samples and associated data for research purposes [17,18,19,20]. Currently, there are hundreds of biobanks worldwide that range from small, predominantly university-based collections, to large, government-supported resources, whose activity, by supporting translational research, is focused on the public interest and aims to provide a public benefit for future generations [6,7,19,20,21,22,23,24,25,26,27,28].

UPO Biobank is the institutional research biobank of the University of Piemonte Orientale (UPO), housed at the Applied Research Centre Ipazia in Novara and integrated with the Regional Research Infrastructure Center of Autoimmune and Allergic Diseases (CAAD).

UPO Biobank came into operation in April 2020, during the first outspread of the COVID-19 pandemic, to meet the need for a systematic and organized collection of samples to sustain research focused on preventive, diagnostic, prognostic, and therapeutic strategies [29,30,31]. In June 2020, once the first COVID-19 emergency was over in Italy [32,33], UPO Biobank was established as a disease- and population-oriented academic research biobank, with a focus on aging research. The UPO Biobank rules and code of ethics have been approved by the Ethics Committee and UPO authorities.

Today, the systematic collection of samples is well underway, in collaboration with local healthcare services, hospitals, and university research groups. In two years of activity, UPO Biobank has collected biological samples and data from more than 1100 subjects, stored up to 35,000 aliquots of human biological samples at +4 °C, −80 °C, and liquid nitrogen, and is emerging as a powerful infrastructure for UPO researchers. Indeed, UPO Biobank provides a rich source of biological materials (i.e., blood and derivatives, saliva, urine, and stool) and the associated data derived from the general population, COVID-19, kidney diseases, diabetes, hematological malignancies, neurological disorders, and autoimmune diseases.

The objective of this manuscript is to report an institutional and operational experience, describing the establishment of a university research biobank with the following purposes: (i) promoting the institution’s quality scientific research with respect for all the parties involved; (ii) supporting cohort studies aimed at promoting and improving health in the reference area; (iii) endorsing public engagement and scientific citizenship actions; and (iv) exploiting samples and data, making them available to the whole scientific community.

## 2. Methods

### 2.1. UPO Biobank Operational Standards

All UPO activities are carried out in accordance with the Ethics Code, a regulation approved by Ethics Committee and UPO Authorities.

High-quality biological material and data are maintained and managed by applying standardized procedures (SOPs) and a quality management system, according to the ISO20387:2019 international standard, developed by the International Organization for Standardization (ISO), a worldwide federation of national standards bodies that work on preparing International Standards through ISO technical committees (www.iso.org/, accessed on 22 November 2022). ISO20387:2019, In particular, ISO20387:2019 specifies the general requirements for biobanking and lists the requirements that biobanks must fulfill to demonstrate their competence in the different aspects of the biobanking operations, as well as their ability to provide high-quality biological samples and associated data for research applications.

### 2.2. Ethical Statement

UPO Biobank code of ethics, regulation, and informed consent have been evaluated and approved by the Ethics Committee of the Ospedale Maggiore della Carità in Novara and by the University of Piemonte Orientale Data Protection Officer (DPO). Studies supported by UPO Biobank have been approved by the competent ethics committees. The prerequisite for enrolment in these studies, as well as the aims and purposes of UPO Biobank, were depicted in the written informed consent.

### 2.3. Sample Management

The human biological samples stored in UPO Biobank are collected, registered, manipulated, and managed following SOPs. Each step of the biobanking process (i.e., sample collection, sample manipulation, sample aliquoting, temporary and long-term storage, release, and transport conditions) is documented in the UPO Biobank SOPs and quality manual, which is available to researchers upon request (UPO_biobank@uniupo.it).

The traceability of biological samples and associated data are assured by an unambiguous, numerical donor identification code and an acronym-specific sample identifier system. Each sample is linked to the associated dataset, whose minimal content is described within the manuscript.

Sample types include whole blood, buffy coat, plasma, serum, peripheral blood mononuclear cells, saliva, urine, and stool. Samples are stored based on the optimal conditions reported in literature and international standards and guidelines at different storage temperatures (i.e., +4 °C, −80 °C, and liquid nitrogen).

### 2.4. Quality Management

The UPO Biobank quality management system involves documented quality control (QC) policies of the biobanking process and SOPs that ensure the monitoring of collection, manipulation, aliquotation, preservation, and distribution of biological material and associated data. Non-conformities in any of these phases are recorded.

The hemolysis grade of plasma and serum samples is evaluated during the processing by assigning a growing value from 0 (none) to 3 (highly hemolyzed). UPO Biobank is setting up routine QC on plasma, serum, peripheral blood mononuclear cells (PBMC), and DNA. Indeed, to monitor the quality of the stored material over time, plasma, serum, PBMC, and genomic DNA are going to be processed and stored for this purpose following SOPs (QC samples). Selected analytes (e.g., reactive C protein, glutamic pyruvic transaminase, alanine transaminase, alkaline phosphatase, thyroid-stimulating hormone, transferrin, fibrinogen, D-dimer, and total proteins) are evaluated on QC plasma and serum samples at the time of collection and, subsequently, once a year. PBMC viability is evaluated before storage by Trypan Blue staining and automated cell counting (Luna II, Logos Biosystems) and recorded. A cell viability control is going to be carried out annually on QC PBMC samples. DNA concentration and purity are determined by measuring the absorbance at 260 and 280 nm in a spectrophotometer (NanoDrop™).

The correct allocation of the aliquots in the cryogenic devices is checked biannually. The UPO Biobank Quality Manager simulates the research and retrieval of 10 to 30 different, random aliquots of biological material by consulting the REDCap database and checking their effective positioning in the devices. In the event of discordances, the malposition is reported as a non-conformity and treated accordingly. Similarly, the associated data are checked biannually. The UPO Biobank Data Manager simulates the research and retrieval of the associated data of 10 to 30 different subjects on the REDCap database and checks their consistency, reporting, and treating the non-conformity derived by a possible discordance.

### 2.5. Data Analysis

All UPO Biobank data are stored in the REDCap database (REDCap, Vanderbilt University). Data presented in this paper were derived from the REDCap database and analyzed using the GraphPad Prism software, version 8 (GraphPad Software Inc., San Diego, CA, USA). Data were presented as numbers and percentages.

## 3. Results

### 3.1. UPO Biobank Finalities

UPO Biobank has been established as a multidisciplinary research biobank with both a population- and disease-oriented commitment, with the aim of promoting studies finalized at exploiting knowledge on human health and encouraging multidisciplinary scientific research on aging. The research areas and objectives of the biobank are clearly outlined in the biobank regulations and, for a consent to be clearly informed, in the UPO Biobank broad informed consent.

As a disease-oriented biobank, UPO Biobank promotes research on human diseases to improve prevention, diagnosis, and therapy within specific areas of interest, such as infection, autoimmune disorders, and high-impact, chronic, age-associated diseases, including cardiovascular, metabolic, neurodegenerative, and neoplastic diseases. Patients participating in disease-specific project biobanking are also asked to participate in the broader aims of the biobank by providing a specific broad consent (discussed later in this paper).

Based on this biobank initiative, disease-specific biobanking studies have already been published (Table 1) [34,35,36,37,38,39,40], and all the invited patients participating in these studies also adhered to the broader aims of the biobank by providing specific consent.

As a population-oriented biobank, UPO Biobank supports prospective and cross-sectional epidemiological cohort studies involving citizens with specific characteristics or who are representative of a geographical area, designed to address both social and scientific unmet needs on human health. In particular, at present, UPO Biobank is engaged in the Novara Cohort Study (NCS), a longitudinal population study aimed at investigating the determinants of longevity and promoting healthy aging in the Novara area.

UPO Biobank is also a non-profit public academic institution and a “service unit” (BBMRI-ERIC:ID:IT_1611942116226242), whose mission is characterized by three main purposes: (1) to encourage and increase a collaborative network between universities, bodies, and associations dealing with public health, in particular hospitals and territorial competent local health services; (2) to encourage the training of university students, so that they are initiated into rigorous research on a scientific level and respectful of ethical principles; and (3) to promote a model of active scientific citizenship, by enhancing citizens’ involvement in public health research and social measures and by constructing communicative strategies able to promote the active, responsible and critical participation of citizens, local institutions and relevant stakeholders in the building of a shared scientific information and awareness.

### 3.2. The UPO Biobank Facility

UPO Biobank is an academic infrastructure consisting of three interconnected facilities: the consulting room, the processing laboratory, and the cryogenic room. Being located in the same building, the three facilities represent a core, integrated biobank structure that supports all of the biobanking-related activities, from the collection of data and biological samples to their manipulation, preservation, and release.

The consulting room is a key facility to conduct population- and disease-oriented studies supported and coordinated by UPO Biobank, as well as a valuable service for studies carried out by independent research groups and destined for biobanking. The room is equipped for the evaluation of functional and anthropometric parameters and for the collection of biological materials, providing both proper privacy and connected services (e.g., private and dedicated toilette). Moreover, an adjacent reserved space has been arranged to meet participants for questionnaire administration and data collection.

The processing laboratory is dedicated to sample manipulation, aliquoting, and analysis. Each operation is carried out following specific and detailed SOPs, and the tracking of samples and aliquots is assured by a multiparametric codification via sample-associated codes and barcodes uploaded in the UPO Biobank database.

The management and traceability of the biobanked samples have proven to be demanding, with a real risk of mistakes and failure [41]. At the beginning of UPO Biobank activity, an in-house adapted system based on the REDCap database (REDCap, Vanderbilt University) was built to accommodate and track the storage of biological samples, as well as the pre-collection and post-storage operations (e.g., study management, informed consent, sample release, etc.). However, the growing demand for these activities prompted the acquisition of a commercial Laboratory Information Management System (LIMS) oriented and structured to answer to the unique needs of biobanking, which will be implemented in the next few months.

The cryogenic room was built following the specific indications for this kind of facility (i.e., ISO11827:2021). It houses 4 °C and −80 °C mechanical freezers and liquid nitrogen tanks connected to an automated system for nitrogen refill. The biobank can accommodate up to 200,000 samples at −80 °C, and up to 500,000 samples in liquid nitrogen.

Since UPO Biobank was implemented with the aim of supporting large population studies, storage capacity was a key issue from the beginning of the UPO Biobank activity planning. In this perspective, the cryogenic room has been equipped with nitrogen tanks fitted to contain straws, doubling their capacity with respect to the classical storage in cryoboxes. This implementation has been linked to an improvement in the processing of the samples by adopting a semi-automated aliquoting system for fluid samples (i.e., plasma and serum), consisting of two MAPI2 machines (Cryo Bio System, L’Aigle, France) that fill, seal, and label the straws.

The samples, collected following specific SOPs, typically include whole blood and its fractions, extracted genomic DNA, whole cell RNA, extracellular vesicles, urine, saliva, and stool. The standardization of the processes has been further implemented by adopting a programmed abatement system for live-cell cryopreservation (Kryo 560-16, Planer), a key instrument for live-cell biobanking that is also suitable for the preservation of cells for cell-based therapy [42,43,44,45].

Access to the cryogenic room is only allowed for authorized personnel through electronic badges and a video surveillance system. The management of the bank is supported by a centralized control system capable of monitoring and recording all the operating parameters (the percentage of ambient oxygen, the liquid nitrogen supply, and the temperature of the cryo-containers) for 24/24 h and 7/7 days. Critical alarms are sent via SMS, voice call, or email to operators for intervention in the event of an emergency. The “life” of each biological sample, from acceptance to analysis, including storage positions, movements, and possible criticalities, is traced by the tracking system and transferred to LIMS.

### 3.3. Building an Efficient Biobank: Networking, Quality Management System, and Certification

The “Biobanking and Biomolecular Resources Research Infrastructure—European Research Infrastructure Consortium” (BBMRI-ERIC) is a pan-European non-profit network of biobanks aimed to improve the accessibility and interoperability of the biobanks of the network as well as to prompt and support the harmonization of the different aspects of the biobanking process [46,47,48]. UPO Biobank joined the national node of the network (BBMRI.it) in February 2021, heading from the beginning toward both an efficient standardization of activity and an incisive interaction between national and European partners. BBMRI-ERIC eases the interaction between biobanks and biobank users by enabling access to the collection of partner biobanks, as well as by supplying expertise and services (i.e., ELSI common service and IT common service). The effective harmonization and interaction among biobanks and between biobanks and final users is a key goal to achieve in the biobanking field, and BBMRI-ERIC, also playing at a national level, is a major promoter of this implementation.

UPO Biobank also adopts strategies to enhance and maintain the quality of the biological samples, starting from their handling to their final preservation, as well as to ensure data protection. Toward this aim, UPO Biobank runs a quality management system that is in agreement with national and international indications [34]. In particular, the quality management system of a biobank contains documented quality control policies and written standard operating procedures underlying the collection, manipulation, aliquotation, preservation, and distribution of biological material and associated data [49,50,51]. The whole biobanking process, from the acquisition to the storage and release of biological specimens and data, is based on Standard Operating Procedures (SOPs) and on the application of guidelines for the equipment used (e.g., sample processing, semi-automated aliquoting, databases, etc.). The UPO Biobank Quality Manager prepares, supervises, updates, and makes available the latest version of all documents, including instructions and authorizations for staff members, control system documents, and SOPs. Following joining BBMRI.it, UPO Biobank is about to complete the internal adaptation for the biobanking-specific ISO20387:2019 certification, which is expected to be achieved by mid-2023.

### 3.4. Sample Storage and Usage: Looking Forward to New Applications

The worldwide spread and growth of biobanks has brought to light the critical aspect of the cost–benefit balance between sample handling and preservation over time, which takes into account the fitness for purpose of the stored samples for future research applications, along with the burdensome and constantly growing employment of physical space and dedicated equipment. However, it is hardly reliable to be able to foresee every future request and application of the biobanked samples. An important aspect of a biobank’s governance and framework is thus the definition of the sample types and the number of aliquots/samples to be collected and stored, taking into account the storage cost over time and the possible effective research applications of the collected samples and data. The key point is that it is not mandatory to collect a huge panel of biological materials but to be able to provide exhaustive documentation of the entire life cycle of the biobanked samples and data, from donor to release, ensuring a sufficient level of adequacy for research purposes. With a view of optimizing the availability of samples and their suitability for future omics investigations, UPO Biobank stores blood and plasma samples collected in the presence of EDTA, useful for proteomic, lipidomic, and genomic analysis [52,53,54], and in the presence of sodium citrate, useful for the downstream purification and analysis of extracellular vesicles (EVs) [55]. Likewise, urine is preserved in large aliquots and pellets, suitable for EV isolation and marker identification [56,57]. Finally, saliva collection optimized with Salivette^®^, a device that allows an easy and fast collection of saliva while avoiding spitting, can be successfully used for different downstream applications [58,59,60].

### 3.5. Access to UPO Biobank

Aiming for a participatory governance model, UPO Biobank encourages the direct interaction between the biobank and stakeholders with the purpose of facilitating useful communication to highlight their needs and expectations and to achieve a fruitful collaboration. To this end, on the website https://biobank.uniupo.it/ (accessed on 24 November 2022), UPO Biobank provides an area dedicated to both citizens and researchers. On the dedicated pages, citizens can find information about the scientific, ethical, and social relevance of participation in biobank activities, the rights of the participants, and an application form to participate. The researcher-dedicated web site area reports all the relevant information about biobanking and how to access samples/data. An inquiry form that can be filled out online and directly sent to the biobank allows researchers to receive information about the following: (i) the availability of specific samples/data, and (ii) the application for a new biobanking project.

UPO Biobank’s staff takes care of answering inquiries within 96 h after receipt and provides assistance for accessing the resources of the biobank or for a new project proposal. All requests are evaluated by the UPO Biobank Technical-Scientific Committee and approved or rejected according to the UPO Biobank Regulation and Ethics Code (which are available online). In all cases, projects supported by UPO Biobank must be approved by the competent Ethics Committee.

The detailed procedures and documents for requesting information can be found on the biobank website (https://biobank.uniupo.it/per-i-ricercatori/, accessed on 24 November 2022). At the moment, the site is only in Italian, but an English version will be available soon.

### 3.6. Biobanking and Databanking

The true value of a biological sample is represented by the associated data, including information about the subject who provided the sample and the information obtained through the laboratory analysis of the biospecimens. Traditionally, such information is stored and managed separately from the samples, both for data size and for security, but with the concrete risk that with time, this information can be lost. Steps forward in data storage and management accessibility and security have allowed biobanks to store both samples and data with increasing benefits for the quality and usability of this wealth of information, moving away from the reductive concept of a biobank as a simple biorepository toward a biobank that manages and possibly generates data associated with samples [61,62].

UPO Biobank, in collaboration with the CAAD bioinformatics facility, is implementing the collection of multi-omics, imaging, or other biologic data, and is improving the data-banking capacity and security by opting for cloud storage and backup of the data, in compliance with Article 32 and Article 89 of the General Data Protection Regulation (GDPR), and observing DPO guidelines, as well as the technical implementation and risk minimization measures resulted from the Data Protection Impact Assessment (DPIA).

### 3.7. An Institutional Biobank Supporting Translational Research and Population Studies

As a disease-oriented biobank, in addition to sustaining COVID-19 research, UPO Biobank is primarily finalized to promote and sustain research on high-impact, chronic diseases, including cardiovascular, metabolic, neurodegenerative, and autoimmune diseases. Researchers are supported by UPO Biobank’s staff in the study design, in identifying the most suitable protocols for the collection and treatment of samples according to project-specific requirements, and in the presentation of research protocols to the Ethics Committee. Moreover, UPO Biobank facilitates the access of samples to the UPO core facility for laboratory investigations, including omics analysis.

**Figure 1:**
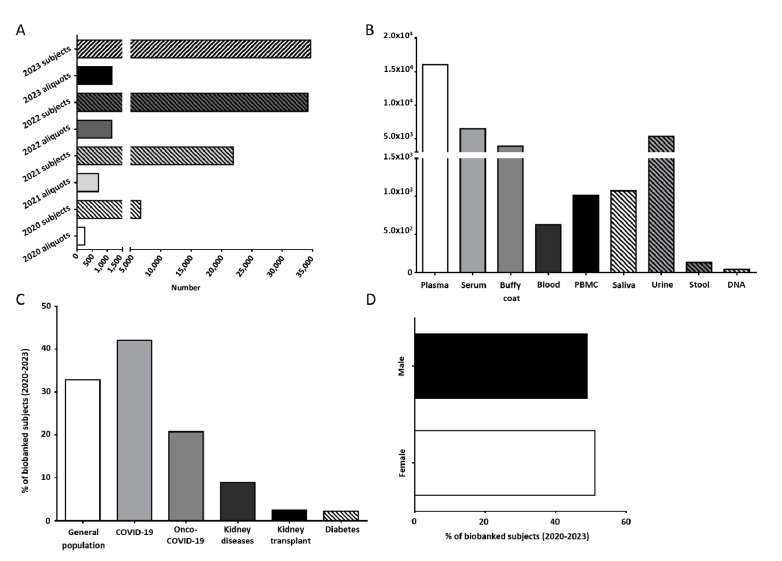
(**A**) Number of subjects and biological samples stored by UPO Biobank from April 2020. (**B**) Number of aliquots of the different biological samples stored in UPO Biobank until today. (**C**) Distribution of the biobanked subjects, highlighting the general population and specific disease-affected subjects. (**D**) Distribution (%) among female and male participants in UPO Biobank.

From its birth in April 2020 until today, UPO Biobank has experienced a gradual increase in the number of supported projects and collected samples (Figure 1A). At present, UPO Biobank hosts samples and data from six concluded research projects and supports seven ongoing ones (Table 1).

At present, UPO Biobank hosts more than 30,000 aliquots of biological samples collected from about 1100 subjects, including whole blood, buffy coat, plasma, serum, peripheral blood mononuclear cells (PBMCs), urine, saliva, stool, and DNA, as shown in Figure 1B. A detailed overview of the samples and associated data actually stored in UPO Biobank are reported in Tables S1 and S2.

About 33% of the samples were collected as part of population studies, whereas nearly 67% of the samples were collected in longitudinal and cross-sectional disease-focused projects, including acute COVID-19 infection and long COVID-19 syndrome, chronic and acute kidney diseases, kidney transplant, and diabetes (Figure 1C).

The age median of the participants is 58 years old (Table S3), with a slightly higher adhesion from female participants with respect to male participants (51.1% vs. 48.9%, respectively, Figure 1D).

From the beginning of the activity, UPO Biobank efficiently supported translational research in UPO, with more than 600 samples released in the past year utilized for three COVID-19-related scientific publications, investigating the response to SARS-CoV-2 infection in the general population and in cancer patients [34,35,36,40], and other projects in progress.

As a population-oriented biobank, UPO Biobank is engaged in the NCS, a longitudinal population study that aims to define the molecular, physiological, and environmental determinants of the trajectories of aging in the area of Novara. The NCS will enroll 10,000 participants older than 35 years old, with a prospective follow-up every 5 years, and the collection of both biological specimens and personal data. For this study, blood, urine, and saliva collection, analysis, and storage were established, creating a multifaceted and constantly growing source of samples and associated data. The biological analysis will contribute to elucidating the molecular mechanism of aging and age-associated diseases. Longitudinal data analysis will also favor the identification of preventive intervention priorities in collaboration with local health authorities. Because of the extensive data and sample collection, the NCS and UPO Biobank will be uniquely rich resources for aging research for all scientific communities.

By supporting the NCS, UPO Biobank will become an integral part of the community, and, in turn, the Novara community will become an extended laboratory. Consequently, the Novara citizens will also find themselves as citizens of the global research community through international research projects.

### 3.8. The Key Role of the Governance

**Figure 2:**
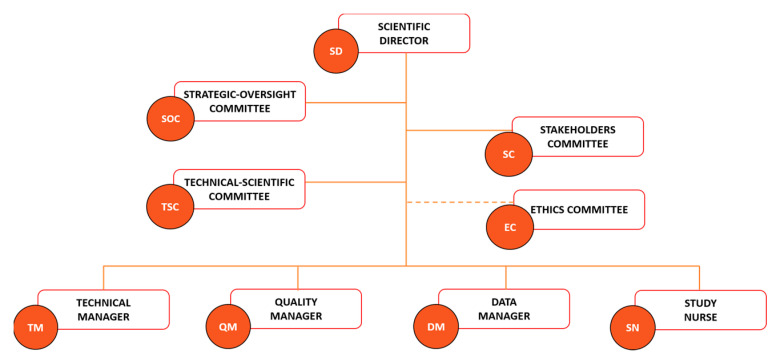
UPO Biobank governance chart.

The legal and ethical aspects of biobanking are key matters in a biobank framework to optimize the use of biobank resources by the scientific community for quality scientific production while respecting the ethics and privacy of the participant. A good governance system based on the three essential pillars of transparency, accountability, and oversight is fundamental for increasing the legitimacy and social engagement of biobanks [63,64]. Particular attention is dedicated to data storage and sharing, with Article 40 of the GDPR encouraging the adoption of a code of conduct aimed at ensuring compliance with the legal requests regarding data processing and privacy [65]. UPO Biobank matched these requirements by adopting a governance system and operational procedures exhaustively described in the biobank regulation, available on the UPO Biobank website [66]. The UPO governance was structured to ensure the engagement of both the scientific community and the population. The Scientific Director (SD), the Strategic Oversight Committee (SOC), and the Technical-Scientific Committee (TSC) (Figure 2) represent the Scientific Board of UPO Biobank, and their role is to direct, lead, and sustain the biobank activity, ensuring the quality of research supported by the biobank’s resources and the respect of the ethical requirements, working together with the competent Ethics Committee. The functioning of biobanking is guaranteed by a Technical Manager (TM), a Data Manager (DM), a Quality Manager (QM), and a Study Nurse (SN).

Biobanks’ activity should be founded on multifaceted perspectives that include both lay stakeholders (e.g., community members and organizations, policy-makers, and patient associations) and field experts (e.g., researchers, clinicians, and ethicists) [63,64,67]. The Stakeholder Committee is a pillar of the UPO Biobank governance chart (Figure 2) and code of conduct, with a scheduled annual meeting focused on the critical evaluation of UPO Biobank activities, aims, and future perspectives. The goal is to achieve constructive communication between UPO Biobank and citizens, ensuring the matching of the stakeholders’ expectations and needs and an interdisciplinary approach to biobanking. These interdependent relationships ensure a deep understanding of the notion and impact of biobanking activity by the lay population, which aims to increase the general approval, trust, and active participation of citizens in future biobanking projects, as well as the safeguarding of the necessities and perspectives of potential biobank’s users, avoiding the low utilization rates of the biobanked material [68,69]. A biobank acts, indeed, as a mediator between the several interests of researchers, clinicians, scientists, participants, and the general population, and must consider all viewpoints in order to make strategic decisions. This participatory governance aims to grant benefit to all the actors of the biobanking process, starting from the participants that entrust the biobank with their biological material and data, allowing new important scientific and health-related discoveries and advancements by the biobank’s customers, which will benefit from a centralized, organized, and certified source of information and material, ensuring in return a concrete and valuable benefit for the participants and the community in general.

### 3.9. Ethical, Legal, and Social Issues

The nature of data processing is a critical issue for the rights and freedoms of subjects, mainly for particular categories of data, such as genetic ones. On this topic, the GDPR, which was adopted by the European Union (EU) in 2016 and came into force in 2018, has proven to be challenging for the biobank community [70,71].

As a recently founded biobank, UPO Biobank immediately faced the integration of GDPR indications into its operational routine. In this regard, data security is a major concern regarding data sharing and usage for research purposes; therefore, before starting the biobanking activity, UPO Biobank carried out the DPIA for the assessment of the impact on data protection, as provided for by Articles 35 and 36 of the GDPR. Indeed, the DPIA is, for all intents and purposes, a mandatory tool to identify and minimize the risks that are inherent to data processing [72,73,74]. The DPIA outcome was constructively applied to improve the data management and security in the UPO Biobank, and it will be updated throughout the lifecycle of the biobank in order to ensure that data protection and privacy are considered and to promote the creation of solutions and compliance.

The GDPR imposes several obligations to scientists, particularly in relation to secondary research uses of personal data (i.e., data used for research studies other than the proposed research that enable biobanking). Biobanks, by implementing the GDPR, together with the technical measures that are necessary to safeguard the rights of data subjects during data processing, can guarantee a profitable use of data for research purposes [69,71,75], but they also face various challenges. The lack of supranational and national bodies with the competence and authority to set uniform and binding requirements causes a certain degree of uncertainty in all the actors involved (researchers, participants, and interested parties) and generates the need for regulations that are specifically dedicated to biobanking [69,71,72,75,76,77]. In Italy, the main reference points are the general authorizations issued by the Italian Data Protection Authority: Authorization no. 8/2016 on the processing of genetic data, and Authorization no. 9/2016 on the processing of personal data for scientific research purposes, but neither of the two authorizations contains provisions directly addressing biobanks [78,79].

Pending an ad hoc legislative act on biobanking, the adaptation for the biobanking-specific ISO20387:2019 certification and accreditation process provides the core elements and guarantees regarding the governance of UPO Biobank in view of GDPR, including conditions for processing personal data, data access models, oversight bodies, and data access and transfer agreements. Furthermore, the adoption of informed consent that clearly exemplifies the research purposes for which the biobanked samples/data and related information will be used, guarantees participants the freedom to decide how their samples will be used.

The social challenges raised by the activity of biobanks are manifold and include the need to adopt a communication strategy that must make use of a shared language, the knowledge and cultural effects resulting from results dissemination, and the cultural and social and not just scientific value of sustainability [80,81,82,83].

UPO Biobank aims to encourage and enhance scientific research by basing its activity on an inclusive model of the scientific community in which citizens, researchers, and institutions will actively participate. For this reason, biological specimens and associated information are recognized as public and institutional resources; the code of ethics and informed consent are deemed institutional documents; and the Stakeholders Committee is a pillar of the UPO Biobank organizational chart [66].

The UPO Biobank’s code of ethics is inspired by the model of “participatory governance” that needs the involvement of citizens, who entrusted biological samples and associated data, researchers, and stakeholders, who support the biobank and have expectations from the biobank’s activities. Starting from individual personal knowledge, this model is based on the shared, intrinsic ability to understand and evaluate technical information, when adequately exposed, in order to participate in public and global decisions [77,84]. By adopting this organizational model, UPO Biobank will pursue the aim of achieving the virtuous circularity that transforms individual contributions into public benefit. The balancing of the potentially divergent needs and interests of these parties is certainly challenging, and the governance system must be proportionate and knowledgeable, especially regarding the risk associated with data sharing and use aimed at producing collective benefits [84,85].

### 3.10. From the Study-Specific Informed Consent toward a Model of Mixed Informed Consent

Starting from the study-specific informed consent, designed to ensure that the participants were deeply informed about the aims and the use of samples and data in the context of a specific and single research, biobanks moved to a new, adapted form of broad informed consent [86,87]. Indeed, although the study-specific consent clearly addresses most ethical issues, this type of consent is not suitable for the purposes of a biobank, which collects biological materials and personal data, especially for long-term future research, which is not clearly defined at present. Moreover, broad consent is less specific than consent for each use, but more narrow than open-ended permission without any limitations (i.e., “blanket” consent).

The broad informed consent approach aims to integrate the specific needs of biobanking while preserving the indisputable protection of the participants [87,88]. This model provides for the gathered general consent when the participant is enrolled and predisposes the future usage of the collected samples for new studies that fulfill the scope stated in the consent, avoiding the necessity to request consent again from the same participants [87,88,89].

The broad model based on general consent is still debated since it is hard to define a priori the future usage and application of the biological material and data collected, and, for consent to be informed, exactly this kind of knowledge is required [89,90,91,92,93]. However, for the purposes of conservation of biological samples, broad informed consent is indicated as appropriate both in the literature and in GDPR recital 33, although the references are fragmentary [90,94].

From these considerations, UPO Biobank and the territorially competent Ethics Committee (for the Ospedale Maggiore della Carità in Novara and the health Agencies of the surrounding area) felt that a “mixed” informed consent, which merges the specific and the broad ones, best suited the biobank aims, a perspective also shared by the Italian Node of BBMRI-ERIC [74]. Indeed, the nature of the consent given by the participants is mixed by nature, since the biobank’s purposes are general but not generic and thrown toward future biobank goals and applications, and in parallel, the information given to the participants about the manipulation and storage of samples and data, as well as their sharing with the scientific community, must be precise and rigorous. Mixed informed consent also presents advantages from an operational point of view. Indeed, by combining the specific research objectives and the broad biobanks’ purposes and aims, integrated with detailed information about personal data processing and protection, mixed informed consent provides a simplified tool intended to facilitate both the request of biobanking from clinicians and researchers and the reading and explanation of the information to the participants. Finally, the broad component of mixed informed consent is a key instrument for explaining and elucidating the general aims and areas of interest of UPO Biobank, ensuring a thorough understanding by the participants of the whole biobanking activity and its impacts. In order to ensure that this type of consent provides protection of autonomy and participant values, a strong ethical review of projects supported by the biobank and continuous communication activity is pursued.

### 3.11. The Interaction with Participants: A Matter of Trust

Although biobanks are widely recognized as a powerful and almost essential tool for biomedical research [19,22,95,96], their activity is not exclusively carried out in clinical and research environments, but necessarily, it relies on the involvement of participants that provide biological samples and information that will sustain multiple research projects.

The unsuccessful enrolment of participants is an actual menace to a biobank’s activity and “trust”, in the view that to confide in biobanks and in those that oversee biobanking to protect participants’ interests, is a matter of utmost importance in the field and remains, indeed, the core value in the relationship between a public biobank and participants [97,98,99]. A major concern for participants is the concept of “personality rights” in relation to how genetic information will be interpreted and used, and who should have access to it, because genomic information is detailed, identifiable in nature, and it affects both the person from whom the information was obtained and the related family members. These are the basic reasons why a biobank, and in particular a population biobank, must match the trust of citizens with the trustworthiness of its governance, as clearly stated by the Organization for Economic Co-Operation and Development (OECD) [100].

UPO Biobank, in conjunction with the launch of the NCS, undertook an intense public awareness campaign through the organization of and participation in public events, institutional stakeholders engagement, mainstream media dissemination, and training interventions among students. A recent survey investigating the level of knowledge and perception of biobanks in UPO students and personnel shows that potential participants were aware of the role that biobanks play in research and were eager to participate for the sake of furthering scientific research. Notably, the study highlights concerns about the confidentiality of the data along with the commercial use of the samples/data [39], encouraging UPO Biobank to disseminate clearer and detailed information about participant rights protection.

### 3.12. Deal with the Challenge: Limitations and Complications in Building an Institutional Biobank

The aspect of sustainability, defined as the maintenance over time of a biobank’s operations and values, has always been a challenge [61,101]. In particular, economic sustainability is a key obstacle to the operability of biobanks over the years, and even though multiple strategies and tools for the cost recovery have been proposed [61,102,103,104], the costs related to the several operational aspects of biobanking exceed the cost-recovery ability of these facilities. On the other hand, biobanks are emerging as pillars for biomedical research and a priority for the European Community, a fact underlined by the integration of biobanks in different framework programs of Horizon 2020, Horizon 2022, and Piano Nazionale di Ripresa e Resilienza (PNRR) funded by Next Generation EU [105,106]. Biobanks’ survival and growth, over time, need both researchers and institutional support. Researchers are called to contribute by designing research projects involving biobanking or using biobanked data/samples and by providing the institution with funding and qualified personnel. As an institutional biobank, UPO biobank is integrated into the academic environment, sharing the typical funding hindrances of this institution. As a non-profit reality, UPO Biobank is working on a cost-recovery plan that aims to cover the operational costs related to biological samples’ management and storage, as well as structure and instrument maintenance. However, in the academic context, effective and long-term planning of their income is challenging, due to the irregular gain of funding. The full integration of biobanks in the academic research routine, foreseeing biobanking costs in grant applications, is the first step to achieve a structured cost recovery. For the future, a primary objective for UPO Biobank will be to cover at least part of the costs of maintenance through the acquisition of external resources. National and international academic collaborations and Horizon 2020 calls represent excellent opportunities, as do partnerships with biotech or pharma companies, always in compliance with the biobank code of ethics.

UPO Biobank infrastructural completion and functionality are supported by the Ministry of Universities and Research (MUR) Department of Excellence funding, received by the Department of Translational Medicine. The budget was spent on sample processing and quality control equipment, the implementation of the storage capacity, and the commercial LIMS. A grant from “Fondo per l’edilizia universitaria e per le grandi attrezzature scientifiche –2020” (MIUR) supported the acquisition of two MAPI2 semi-automated samples aliquoting systems, whereas the increase in storage capacity with the acquisition of an additional nitrogen tank was possible thanks to INFRA-P funding managed by Regione Piemonte (Italy).

The technical management of a biobank’s infrastructure and resources—such as storage facilities and sample/data processing—is central to maintaining quality, and it determines the relevance and success of a biobank, but requires trained personnel. However, the recruitment of dedicated and qualified personnel is a well-known challenge for biobanks. With the aim of guaranteeing a prospect of growth and stability for its research infrastructures, the UPO administration has established a stabilization program for technical staff. In particular, UPO Biobank has been equipped with dedicated trained personnel, represented by a Technical Manager (TM), a Data Manager (DM), a Quality Manager (QM), and a Study Nurse (SN). These figures have clear and defined duties that are critical to the proper functioning, maintenance, and growth of the biobank.

Another important challenge has been ensuring the continuity of UPO Biobank’s activity and monitoring 7/24. Toward this aim, UPO Biobank, in close collaboration with the UPO Innovation, Digitalization, and Process Quality division staff, is defining a Business Continuity Plan, which also includes technical personnel who can intervene when an emergency occurs. It is inevitable that the satisfaction of these needs strongly depends on the resource and personnel management policies of the institution in charge of the biobank. The disaster recovery plan is another delicate issue for Italian biobanks, frequently represented by biorepositories located in small and hard-to-reach buildings. UPO Biobank faced this problem by stipulating a contract with an external, private company located in the north of Italy that ensures to take action within 2 h in the event of malfunctioning of any cryogenic container, taking charge of the biological samples as long as the malfunctioning has been fixed, and maintaining the cold chain and traceability of the material.

A paramount aspect of building an institutional biobank is to focus attention not only on short-term biobank and stakeholders’ objectives, but also on medium- and long-term ones. Indeed, the future implementations of a biobank in terms of physical space, infrastructures, instrumentation, data storage, etc., should be preventively conceived to reduce the future costs and time spent. UPO Biobank, supporting population studies, has forecast a progressive increase in biological materials and associated data contribution to biobanks and has been designed to take the possibility of doubling its initial storage capacity into consideration.

Privacy and data security are pillars of a biobank’s organization, as discussed above. This implies durable cooperation with the institutional figures deputed to privacy and data protection. UPO Biobank foresaw a privacy-by-design approach and immediately worked on an ENISA, a DPIA, and a data protection policy in cooperation with the UPO DPO and Information and Communication Technologies division. Biobanks, to truly support quality research that serves the whole community, need to collect not only biological samples but also associated quality data. Therefore, the need to engage in a dialogue with local health authorities regarding the shared use, within the framework of the GDPR, of health and research data is becoming increasingly pressing. Several Italian biobanks, including UPO Biobank, are moving in this direction with a view to collaborating with regional and national institutions for the pursuit of actions aimed at improving public health and the acquisition of new knowledge that can be placed at the service of the whole community.

## 4. Discussion

Biobanks are powerful tools for biomedical research that represent the core of the scientific process, granting undeniable quality, impartiality, and ethics to scientific results. Academic biobanks, in particular, are integrated into an ideal environment aimed at promoting and sustaining multidisciplinary research that complements social and scientific finalities. By sustaining population studies, such as the Novara Cohort Study, UPO Biobank will lay the foundations for a platform in support of epidemiological and social studies aimed at promoting healthy aging and public engagement in scientific research.

Moreover, UPO Biobank, as an institutional infrastructure, is sustaining the development of a collaborative network between the university and local healthcare services and authorities, including hospitals and local health units. The presence of this collaborative network will allow the implementation and consolidation of a scientific collaboration aimed at promoting health in the territory that can have a tangible impact through the implementation of innovative primary, secondary, and tertiary prevention measures. However, this goal could only be achieved with a constant exchange with the local healthcare services and authorities, together with a shared purpose of integrating biobanking into the clinical routine. UPO Biobank successfully cooperates with different clinical units of the Ospedale Maggiore della Carità in Novara and is working on a communication channel with the local healthcare authority with the purpose of expanding the efficient cooperation at the local and regional levels.

Finally, the current project’s objective, the Excellence Project funded by the Italian Ministry of University and Research (MUR), to implement an information technology (IT) and artificial intelligence infrastructure to support UPO Biobank and NCS, will allow, in the future, a deep and integrated analysis of biobanked data.

## 5. Conclusions

Thanks to the standardized manipulation and preservation practices linked to organized data management, UPO Biobank is emerging as a dynamic and fast-growing facility in UPO with the potential to sustain local biomedical and translational research. As a member of the BBMRI.it network, the development and improvements to UPO Biobank are indeed outward-looking to expand collaboration and encourage the efficient and simple sharing of biological samples and data within the scientific community, in compliance with national and international laws and ethical guides.

Moreover, the UPO biobank is a technological tool for scientific innovation with a potentially strong social and economic impact on the territory. An infrastructure of this value may indeed contribute to R&D with local and international partners and attract further public and private investments in the Novara area.

## Figures and Tables

**Table 1:** Main UPO Biobank biobanking projects.

Study	Description	Collection Period	Number of Participants	Publications
**UPO-COVID-19**	Collection of blood from COVID-19 patients during the very first outspread of the disease in Italy (April–June 2020).	2020	106	/
**UnIRSa Cohort Study** Unveiling the Immune Response to SARS-CoV-2 Infection	Evaluation of the immune response, during the time, to SARS-CoV-2 epitopes in patients recovered from COVID-19 and the efficacy of specific anti-SARS-CoV-2 antibodies as neutralizing agents.	2020–2021	100	Griffante, G. et al., 2021 [36]
**ddPCR OnCOVID** Comparative Analysis of SARS-CoV-2 Molecular Techniques on Different Biological Samples in a Cohort of Patients Undergoing Anticancer Therapy	Investigation of droplet digital PCR (ddPCR) and neutralization assay (NTA) for the management of SARS-CoV-2-infected onco-hematologic patients (i.e., to detect the neutralizing antibodies).	2021	33	Borgogna, C. et al., 2022 [34]
**A.O.U. ddPCR SARS-CoV-2** Viral Surveillance on the Medical Staff of the A.O.U. “Maggiore della Carità” by Droplet Digital PCR Analysis Performed on Saliva Samples	Comparison between oropharyngeal swab and saliva samples for the detection of SARS-CoV-2 infection by ddPCR, with the purpose to better define a sanitary surveillance strategy for the healthcare personnel of the A.O.U. “Maggiore della Carità”.	2021	18	/
**ema-NTA** Immunological Surveillance Protocol to Assess the Effectiveness and Duration of Post-infection Immunity from SARS-CoV-2 in a Cohort of Hematological Patients	Analysis of the humoral immune response in SARS-CoV-2-infected hematological patients, who have had and got over COVID-19 disease with a neutralization assay.	2021–2022	197	Borgogna, C. et al., 2022 [34] Borgogna, C. et al., 2022 [35]
**BioMAge** Pilot Study for the Identification of Biomarkers Signatures of Aging and Longevity	Identification of aging biomarkers that will help to define the individual rate of aging, the risk of illness and death, and the impact of longevity interventions.	2020-ongoing	193	Bettio, V. et al., 2023 [38]
**DM-PREVENT** Novel Intestinal Microbiota-based Medicine for Preventing Type 2 Diabetes Mellitus	Assessment of the effects on insulin sensitivity of the probiotic containing *Intestinimonas butyriciproducens* in adults with prediabetes.	2021-ongoing	28	/
**TED** Prevention of Falls in the Elderly Using the Monitoring System TED	Assessment of the efficacy of the wristband TED in the identification of falling-risk patients affected by Parkinson’s Disease.	2020-ongoing	20	Campani, D. et al., 2022 [37]
**NO-MORE-COVID-19** Early Diagnosis of Comorbidity and Assessment of the Effective Immunization in COVID-19 Patients	Assessment of respiratory, radiological, motor, and psychological sequelae in COVID-19 patients after 1 year from hospital discharge.	2021-ongoing	301	Bellan, M. et al., 2022 [40]
**KETOMI** VLCKD (very-low-calorie ketogenic diet) in patients with type 2 diabetes and non-alcoholic fatty liver steatosis	Investigation of the complex interplay between inflammation, hormones, microbiota composition and functions, and messengers (e.g., extracellular vesicles and metabolites) to give tailored indications in the clinical management of type 2 diabetes and non-alcoholic fatty liver steatosis.	2022-ongoing	3	/
**KT-UPO-B** Creation of a Biobank for the research of biomarkers related to immunological and non-immunological dysfunction of the graft in patients with renal transplant	Collection of biological samples from a large cohort of patients with a renal transplant, with a long systematically documented clinical–laboratory follow-up for the identification and validation of new biomarkers related to the main complications of renal transplantation.	2023-ongoing	2	/
**NCS** Novara Cohort Study	Longitudinal cohort study aimed at the identification of biological, social, and environmental determinants associated with the different trajectories of aging in the Novara area (Italy).	2022-ongoing	56	/
**SIDERALE** Susceptibility to infectious diseases in obese patients: an endocrinolgical, translational and sociological analysis	Finalized to demonstrate the association between oral and intestinal microbiota with the susceptibility to generic infections in obese patients.	2023	/	/
**DELIVIDA** Determination of plasmatic Vitamin D in elderly pulmonic or septic patients	Investigating the association between plasma vitamin D levels and the prognosis in elderly subjects hospitalized for either pneumonia of any origin or sepsis.	2023	/	/
**OPTION** Prospective cohort of patients affected by pulmonary hypertension: discovery of diagnostic and prognostic markers	Evaluating the role of the extracellular vesicles in both pathogenesis and diagnosis of connective tissue-associated pulmonary hypertension.	2023	22	/

## Data Availability

The biological material and associated data stored in UPO Biobank can be obtained following a successful application and evaluation process. If the researcher’s request is accepted by the TSC, a data and material transfer agreement is signed and both biological material and data can be supplied. Further details can be found at https://biobank.uniupo.it/ (accessed on 24 November 2022).

## Supplementary Materials

The following supporting information can be downloaded at: https://www.mdpi.com/article/10.3390/jpm13060911/s1, Table S1: Biological material stored in UPO Biobank; Table S2: Associated data stored in UPO Biobank; Table S3: Age distribution of subjects.
