# Supplementary material for: UPO Biobank: The Challenge of Integrating Biobanking into the Academic Environment to Support Translational Research
Source: J Pers Med. 2023 May 29;13(6):911. doi: 10.3390/jpm13060911 (PMC10304261; doi:10.3390/jpm13060911)
Supplement: Supplementary file 1 [file jpm-13-00911-s001.zip › jpm-2357802-supplementary.pdf]

Supplementary Table S1. Biological material stored in UPO Biobank.

PROJECT	EDTA plasma	Sodium-citrate plasma	Lithium-heparin plasma	Serum	PBMCs	DNA-RNA	Saliva	Urine	Stool
UPO-COVID-19	X	X	X	X	X	X			
The UnIRSA Cohort Study	X	X		X		X	X		
ddPCR OnCOVID	X	X		X		X			
AOU-ddPCR	X	X		X	X	X			
ema – NTA	X	X		X		X			
BioMAge	X	X	X	X		X	X	X	
DM-PREVENT	X	X	X	X		X		X	X
TED	X	X	X	X	X	X			
NO-MORE-COVID-19	X	X	X	X		X	X	X	
KETOMI	X	X	X	X		X		X	X
KT-UPO-B (sampling begins in 2023)	X		X	X	X	X	X	X	

NO-MORE-COVID-19	X	X	X	X		X	X	X	
SIDERALE (sampling begins in 2023)	X	X	X	X		X		X	X
DELIVIDA (sampling begins in 2023)	X	X	X	X	X	X	X	X	
OPTION (sampling begins in 2023)	X	X	X	X		X	X	X	X

Supplementary Table S2. Associated data stored in UPO Biobank.

LABORATORY DATA				
**HEMATOLOGY**	**DIFFERENTIAL LEUKOCYTE COUNT**	**BIOCHEMISTRY**	**URINE BIOCHEMISTRY**	**MOLECULAR BIOLOGY**
• Laboratory location<br>• Reference values<br>• Exam date<br>• White blood cells (x10^3/microL)<br>• Red blood cells (x10^6/microL)<br>• Hemoglobin (g/dL)<br>• Platelets (x10^3/microL)	• Neutrophils %<br>• Lymphocytes %<br>• Monocytes %<br>• Eosinophils %<br>• Basophils %	• Basal glucose (mg/dl)<br>• Creatinine (mg/dl)<br>• Estimated glomerular filtrate rate (eGFR)<br>• Total cholesterol (mg/dl)<br>• Cholesterol HDL (mg/dL)<br>• Cholesterol LDL (mg/dL)<br>• Triglycerides (mg/dl)<br>• GOT/AST (U/L)<br>• GPT/ALT (U/L)<br>• GGT (U/L)<br>• Glycated hemoglobin<br>• Na (mEq/L)<br>• K (mEq/L)	• Morphologic description<br>• pH<br>• Glucose (mg/dL)<br>• Proteins (mg/dL)<br>• Hemoglobin (mg/dL)<br>• Ketone bodies (mg/dL)<br>• Bilirubin (mg/dL)<br>• Urobilinogen (mg/dL)<br>• Specific weight<br>• Nitrites<br>• Leukocyte esterase (Leu/microL)	• Research of SARS-CoV2<br>• Date 1st + swab<br>• Date 1st – swab<br>• IgG SARS-CoV-2 antibodies (CLIA method)<br>• Serology date

ANAMNESIS				
ANAMNESIS	GENERAL INFORMATION	PATHOLOGIES	ACTIVITIES - OCCUPATION	WORK
	• Screening programs (breast, colon, rectum, etc.)<br>• Surgical interventions (nervous, endocrine, respiratory system, etc.)	• Type of pathology (Alzheimer, hepatic cirrhosis, depression, etc.)<br>• Date of diagnosis<br>• Details on the kind of disease<br>• Stage of the disease<br>• Medicines/homeopathic products	• Employment definition (full-time, part-time, unemployed, retired, etc.)<br>• Work shifts<br>• Physical activity performed in the workplace (sedentary, standing, manual, etc.)<br>• Exposures (solvents, varnishes, paints, etc.)	• Work category (agriculture, industry, mechanical engineering, transportation, etc.)<br>• Clarifications about the workplace<br>• Clarifications on the instrumentations and materials used

Supplementary Table S3. Age distribution of subjects.

AGE - MINIMUM	AGE - MAXIMUM	AGE – MEAN	STANDARD DEVIATION	AGE - PERCENTILE						
				0.05	0.10	0.25	0.50 (MEDIAN)	0.75	0.90	0.95
21	92	57	16.01	81	77	69	58	45	32	28
